# Effect of blood flow restriction training on health promotion in middle-aged and elderly women: a systematic review and meta-analysis

**DOI:** 10.3389/fphys.2024.1392483

**Published:** 2024-07-02

**Authors:** Mengyun Feng, Jian Li, Jinzhen Zhao, Xianqi Pan, Mengyu Wang, Qi Han

**Affiliations:** ^1^ China Ice Sport College, Beijing Sport University, Beijing, China; ^2^ College of Sports Coaching, Beijing Sport University, Beijing, China; ^3^ Sports Nutrition Center, National Institute of Sports Medicine, Beijing, China

**Keywords:** blood flow restriction, KAATSU, elderly women, BFR, health

## Abstract

**Background:** Physical activities play an important role in alleviating the aging problem and improving the physical fitness of middle-aged and elderly people. Blood flow restriction (BFR) training, also known as pressure training, has been widely used to improve athletes’ performance and rehabilitation, which is a relatively novel exercise method for improving the physical fitness of middle-aged and elderly people. The purpose of this study is to conduct a systematic review and meta-analysis of domestic and foreign randomized controlled trial studies on BFR training for middle-aged and elderly women, further explore the impact of BFR training on health status.

**Methods:** Meta-analysis was performed according to PRISMA standards, and charts were drawn using Review Manager 5.4 and Stata 17 software. In this study, the keywords such as “pressure training”, “blood restriction training”, “elderly women”, “KAATSU”, “blood flow restriction training” were used on CNKI, China Science and Technology Journal Database, PubMed, Embase, Web of Science, Cochrane Library, EBSCO, Scopus, and randomized controlled trials were searched in all languages. The search was performed from the establishment of database to 2 January 2024. The results of the combined effect were represented by standard mean differences.

**Results:** Among the 681 literature retrieved, six eligible English articles were included in this meta-analysis. The overall effect test of the combined effect was performed on 10 groups of data, and the results were SMD = −0.18 (95%CI: −0.91 to 0.56; *p* > 0.05), the maximum dynamic force of 1RM SMD = 0.97 (95%CI: 0.35 to 1.58; *p* < 0.05), leg compression force SMD = −0.10 (95%CI: −0.78 to 0.57; *p* > 0.05), heart rate SMD = 0.33 (95%CI: −2.50 to 3.17; *p* > 0.05), systolic blood pressure (SBP) SMD = −1.44 (95%CI: −2.17 to −0.70; *p* < 0.05), diastolic blood pressure (DBP) SMD = −0.69 (95%CI: 2.54 to 1.15; *p* > 0.05).

**Conclusion:** BFR training had a significant effect on the increase of the maximum dynamic force of 1RM and decrease of blood pressure in middle-aged and elderly women, but there was no significant difference found in heart rate and leg compression force.

**Systematic Review Registration:** https://www.crd.york.ac.uk/prospero/, identifier CRD42024491642.

## 1 Introduction

In 2022, the World Health Organization pointed out that at present, all countries are facing the important problem of population aging, and by the end of 2022, China’s population aged 60 and above will reach 280.04 million, accounting for 19.8% of the total population (Linlin, 2023). With the development of economy, the aging problem of society is becoming more and more serious, and the occurrence probability of chronic diseases among middle-aged and elderly women in China is also greatly increased (Wang et al., 2019). Aging will lead to a large loss of muscle and bone components in the body, musculoskeletal dysfunction and cardiovascular disease are the most common chronic diseases. Physical exercise can improve the physical function of middle-aged and elderly people (Dipietro et al., 2019; Amato et al., 2022), appropriate exercise can improve muscle strength, respiratory system function (Agostini et al., 2023), reduce the incidence of chronic diseases and improve the physical fitness for middle-aged and elderly people (Guoqiang, 2006). Middle-aged and elderly women have high limitations in the choice of sports mode due to their own functional status, especially middle-aged and elderly women with osteoporosis and hypertension. In traditional high-intensity training, the loading intensity that promotes strength growth is close to 60%–80% of 1RM (Hunter et al., 2004), which is easy to cause injury and a suddenly increase in blood pressure (Carroll et al., 1992; Kallinen and Markku, 1995). For middle-aged and elderly women, it is impractical to increase strength by increasing training load, resistance or training volume, and there are many experiments proved that the effect of high-intensity exercise can be achieved through BFR combined with low-intensity exercise (Thiebaud et al., 2013; Vechin et al., 2015; Mânica et al., 2020). Therefore, it is very important to explore the suitable exercise mode for middle-aged and elderly women.

In the 1960s, Japanese sports doctor Sato (2005) first proposed the application of blood flow restriction (BFR) to sports training, the initial BFR training is the use of bicycle inner tubes tied to the limb parts of training, with the extensively increased application of BFR training, the equipment is becoming more and more advanced and efficient. BFR training, also known as pressure training, refers to the use of specific tools to compress the proximal end of the limb and block blood flow within a certain range, during which time, low-intensity BFR training can stimulate muscle growth. Now, it is widely used to increase muscle mass and strength, improve aerobic capacity, prevent disused muscular atrophy, and accelerate the recovery process after injury and surgery (Wei Jia et al., 2019).

BFR exercise may be an effective exercise method for improving the health status of middle-aged and elderly women, but there are few studies on the effectiveness of BFR training for middle-aged and elderly women, and whether it can promote their health is still controversial. Some studies suggest that BFR training may cause abnormal cardiovascular reactions (Iida et al., 2011; Ozaki et al., 2011; Santler and Goerge, 2017; Cristina-Oliveira et al., 2020), and the safety of BFR training needs to be studied (Spranger et al., 2015). Although it has some shortcomings/weakness, most experts still take BFR training and find that it can quickly and effectively enhance the physique of the elderly (Clarkson et al., 2017; Barili et al., 2018; Letieri et al., 2019; Kargaran et al., 2021; Maciel et al., 2021; Xiong and Liu, 2023). In this study, meta-analysis was used to integrate the results of BFR training for middle-aged and elderly women, and on the basis of collecting various indicators affecting muscle shape, blood pressure and strength, quantitative analysis was conducted to verify its effectiveness and reliability of the combined results from current research.

## 2 Materials and methods

### 2.1 Study and registration

This review is registered at PROSPERO (Registry Information) and was written in accordance with the standards for systematic reviews and meta-analyses published by PRISMA (Registration number: CRD42024491642).

### 2.2 Eligibility criteria

#### 2.2.1 Inclusion criteria

The inclusion criteria used in this analysis were: 1) The study type was randomized controlled trial (RCT); 2) BFR intervention was performed in the experimental group; 3) The subjects were middle-aged and elderly women over 55 years old, and there was no significant difference in their baseline characteristics; 4) Muscle strength, muscle mass and heart rate were included in the results, and data were expressed as mean ± standard deviation (SD); 5) Include sufficient data to calculate the effect size; 6) The intervention intensity and duration of BFR are not restricted.

#### 2.2.2 Exclusion criteria

The literature will be excluded if: 1) Repetitive literature; 2) There is a copyright problem or the full text cannot be obtained; 3) The data in the paper are fuzzy, the outcome indicators and data are incomplete; 4) Research design errors; 5) Use of other drugs or change the subjects' medication habits.

### 2.3 Literature search strategy

To identify eligible studies, literature searches were conducted in Chinese and English databases including CNKI, China Science and Technology Journal Database, PubMed, Embase, Web of science, Cochrane Library, EBSCO and Scopus from inception to 2 January 2024 (search deadline). It is not limited by regions, publication types or languages.

The search terms used were pressure training, blood restriction training, blood obstruction training, elderly women; KAATSU, KAATSU training, blood flow restriction training, BFR therapy, BFRT, occlusion training, blood flow restriction with exercise, resistance training associated with blood flow restriction, resistance training associated with BFR, elderly women. Each synonym is retrieved using the “or” connection. The search type is: “KAATSU or KAATSU training or blood flow restriction training or BFR therapy or BFRT or occlusion training or blood flow restriction with exercise or resistance training associated with blood flow restriction or resistance training associated with BFR” and elderly women.

### 2.4 Study selection and data extraction

In the first screening, the titles, abstracts and keywords of the relevant literature retrieved were browsed and evaluated according to the inclusion criteria, and the literature that do not meet the criteria are excluded. Screening with Zotero software was applied to remove duplicate entries. The second screening determining the final literature based on the full text and the systematic exclusion of studies that did not meet the inclusion criteria. Data extraction was carried out independently by two researchers, and any differences were resolved by a third researcher or by consensus. From the eligible literature, we extracted the following relevant information: 1) The name of the first author; 2) The year of publication; 3) The comparison result datasets between the experimental group and all control groups without BFR training; 4) Training scheme, frequency and time of experimental group and control group; 5) BFR group cuff pressure; 6) Outcomes: muscle strength, blood pressure, heart rate. All the relevant datasets were extracted and calculated in a spreadsheet Table 1.

**TABLE 1:** Sensitivity analysis of included references.

Rejection sequence number	*p*	I^2^^(%)^	SMD, 95%CI
1	*p* < 0.001	78	−0.39 [−1.08, 0.31]
2	*p* < 0.001	80	−0.01 [−0.75, 0.72]
3	*p*< 0.001	80	0.02 [−0.75, 0.72]
4	*p*< 0.001	84	−0.19 [−1.03, 0.64]
5	*p* < 0.001	84	−0.01 [−1.04, 0.60]
6	*p* < 0.001	83	−0.06 [−0.84, 0.71]
7	*p* < 0.001	83	−0.07 [−0.86, 0.71]
8	*p* < 0.001	81	−0.31 [−1.09, 0.46]
9	*p* < 0.001	83	−0.30 [−1.08, 0.47]
10	*p* < 0.001	84	−0.17 [−0.99, 0.64]

### 2.5 Risk of bias in studies

The risk of bias was assessed in accordance with the Cochrane Manual for Systematic Review of Interventions, and each study was individually evaluated using seven criteria to assess the risk of bias. Evaluation was conducted independently by two investigators from random sequence generation, assignment method concealment, implementation bias, measurement bias, follow-up bias, selective reporting of findings, and other sources of bias. For each trial, we assessed and graded the risk as low, possibly low, unclear, possibly high, or high bias risk. If the risk of bias is low or likely to be low in all areas, the overall risk of bias is classified as low; if the risk of bias is not clear for the above risks of bias, it is graded as not clear risk; high if the risk of bias is high or likely to be high when it has at least one risk of bias. Disputes are handled by a third researcher or through discussion and negotiation before a decision is made.

### 2.6 Meta-analysis

Meta-data analysis was conducted independently by two authors. Basic information of subjects was extracted in the form of mean value, standard deviation (SD) and sample size. Baseline values and post-treatment values were used for meta-analysis. The software Review Manager 5.4 and Stata 17 were used to process the data and generate forest as well as funnel plots. In this paper, continuous result variables are used, and plots are represented by standard mean differences (SMD). Heterogeneity exists when *P* ≤ 0.1 or I^2^ ≥ 50% for the overall effect analysis. When there was no heterogeneity, the fixed effect model was used. When heterogeneity was present, random effects model was applied. Subgroup analysis or regression analysis and sensitivity analysis were used to explore the sources of heterogeneity, forest plots were used to assess different impacts between intervention and control, and funnel plots were used to analyze publication bias. *p* < 0.05 was considered statistically significant.

## 3 Results

### 3.1 Study selection

**FIGURE 1:**
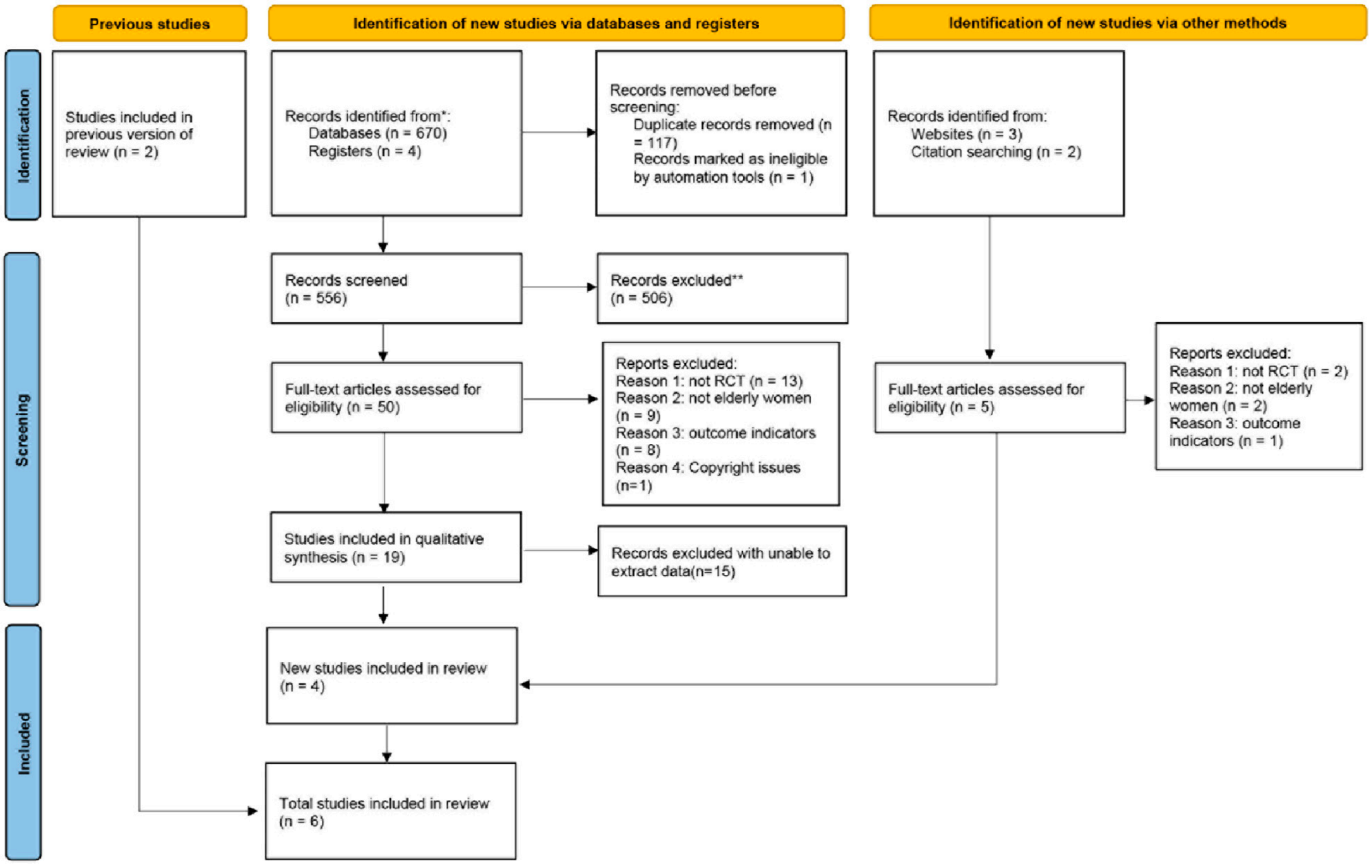
Literature screening process.

A total of 681 articles were retrieved in the database, and the literature were imported into Zotero, a literature management software. After removing duplicate records and multiple screening, 21 articles were found to meet the inclusion criteria. Due to the reasons of data acquisition, six eligible English articles were finally included in the study. Figure 1 shows the literature screening process.

### 3.2 Study characteristics

The six eligible trials included a total of 129 elderly women over 55 years of age, 48 in the experimental group and 81 in the control group.

Table 2 shows the features of all included studies. The experimental group took BFR training, while, the control group did not perform any type of BFR intervention. Muscle strength, heart rate and blood pressure are taken as outcome indicators to analyze whether they can promote the health of elderly women.

**TABLE 2:** Features of all included studies.

Study, year of publication	Country	Year of study	Age (years)	N	Protocol	Intervention				Indicators	Conclusion
						Strength (% 1RM)	Duration (week)	Training scheme	BFR group cuff pressure (mmHg)		
Silva et al. (2015)	Brazil	2015	62.2 ± 4.53	5	LI + BFR	30	12 weeks (2 days/week)	Four sets with an average of 7.0 ± 3.38 repetitions in each group and an interval of 30 s	104.20 ± 7.88 (mean pressure)	Muscle strength	Low-intensity ST combined with BFR seems to be effective for increasing MDS in elderly women with osteoporosis
				5	HI	80		Four sets with an average of 8.0 ± 2.01 repetitions in each group and an interval of 2 min			
				5	CON	ND		ND			
Ruaro et al. (2019)	Brazil	2019	64.69 ± 3.74	17	LL-BFR	40	14 weeks (2 days/week)	Three sets of 15 repetitions with a 40 s interval between sets	70% of systolic blood pressure	Muscle strength	It was concluded that the exercise of wrist flexion (at 40% 1RM) with BFR performed immediately before a traditional RT session (70% 1RM) were able to produce greater gains in maximal dynamic force and lower limb functional strength than RT alone
			67.12 ± 4.97	16	HL	70					
Linero and Choi (2021)	South Korea	2021	56 ± 1.8	7	MHIRT	60–80	12 weeks (3 days/week)	Three sets of 10 repetitions with a 60 s interval between sets	188 ± 9 (mean pressure)	Muscle strength and Muscle mass	LIBFR can be to improve muscle strength
				7	LI-BFR	30		Three sets of 20 repetitions with a 30 s interval between sets			
				6	LIRT	30		Three sets of 20 repetitions with a 30 s interval between sets			
				6	CON	ND		ND			
Thiebaud et al. (2013)	United States	2013	61 ± 5	8	MH	70–90	8 weeks (3 days/week)	Three sets of 10 repetitions with a minute or two interval between sets	80 (the first week); 90 (the second week); 100 (the third week); 110–120 (the fourth week); 120 (from weeks 5–8)	Muscle strength and Muscle mass	moderate-to-high-intensity EB training and low-intensity EB training with BFR resulted in similar increases in strength, total bone-free lean body mass and muscle thickness
				6	LI-BFR	10–30		One set of 30 repetitions and two sets of 15 repetitions with a 30 s interval between sets			
Cezar et al. (2016)	Brazil	2016	63.75 ± 11.58	8	LIRT	30	8 weeks (2 days/week)	Three sets with loads corresponding to 30% of their 1-RM strength at intervals of 30-s between sets	70% of the subject’s resting systolic pressure	Cardiovascular system: blood pressure, heart rate	Resistance exercise during 8 weeks was effective in lowering blood pressure in medicated hypertensive subjects
				8	HI	80					
				7	CON	ND					
Pinto et al. (2016)	United States	2016	67.0 ± 1.7	6	BFR	20	Acute	Three sets of 10 repetitions an interval of 1 min	80% of the necessary pressure for full blood interruption	Cardiovascular system: blood pressure, heart rate	In comparison with high-intensity resistance exercise, low-intensity resistance exercise with BFR can elicit: (i) same haemodynamic values during exercise; (ii) lower rating of perceived exertion; (iii) lower blood lactate; (iv) higher haemodynamic demand during the rest interval
				6	without BFR	65					
				6	CON	ND					

LI + BFR, low-intensity strength training with BFR; HI, high intensity exercise; LL-BFR, 40% 1RM with BFR; HL, 70% 1RM without BFR; MHIRT, High-Intensity resistance training; LIRT, low-intensity resistance training; MH, a moderate-to high-intensity elastic band training group; LI-BFR, a low-intensity elastic band group training combined with BFR; RM, repetition maximum; CON, controlled group; ND, no description.

### 3.3 Risk of bias in studies

**FIGURE 2:**
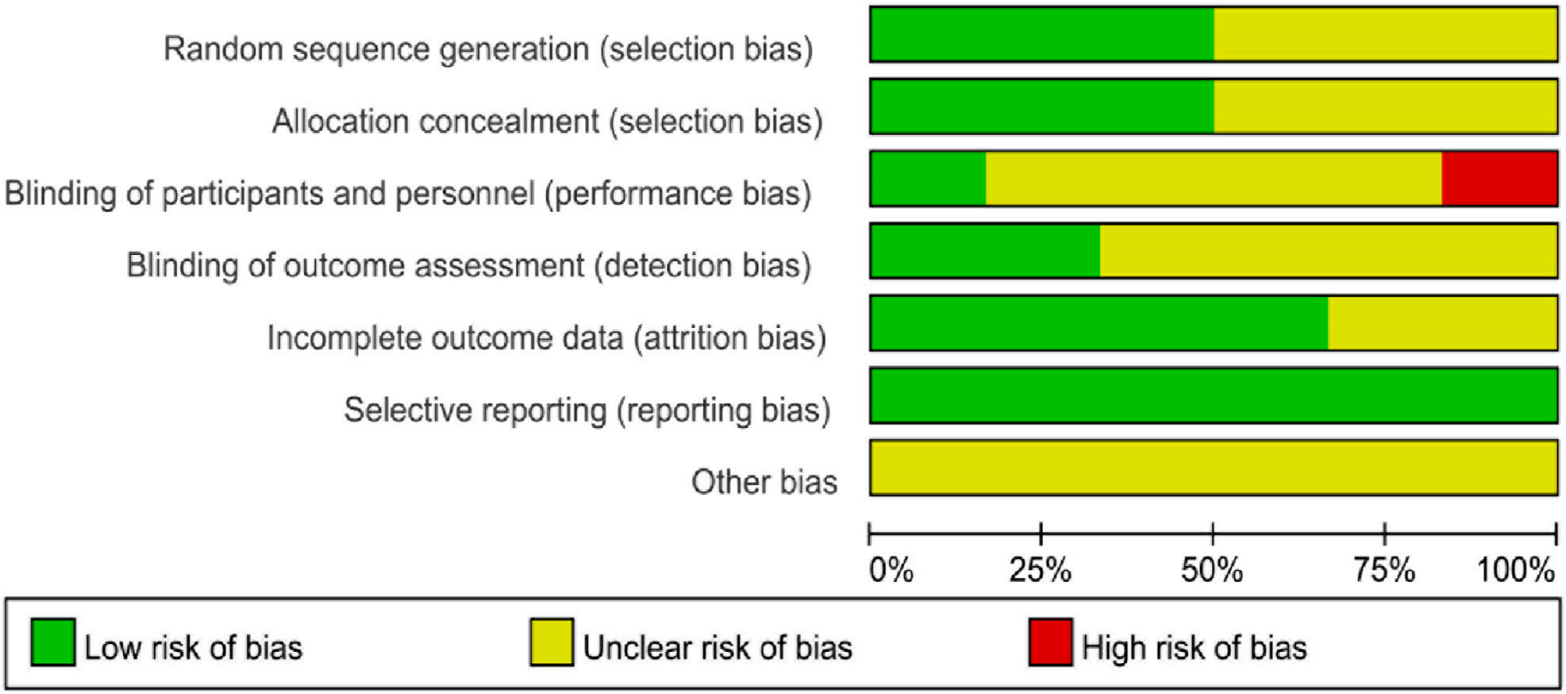
Risk assessment diagram of bias in included studies.

**FIGURE 3:**
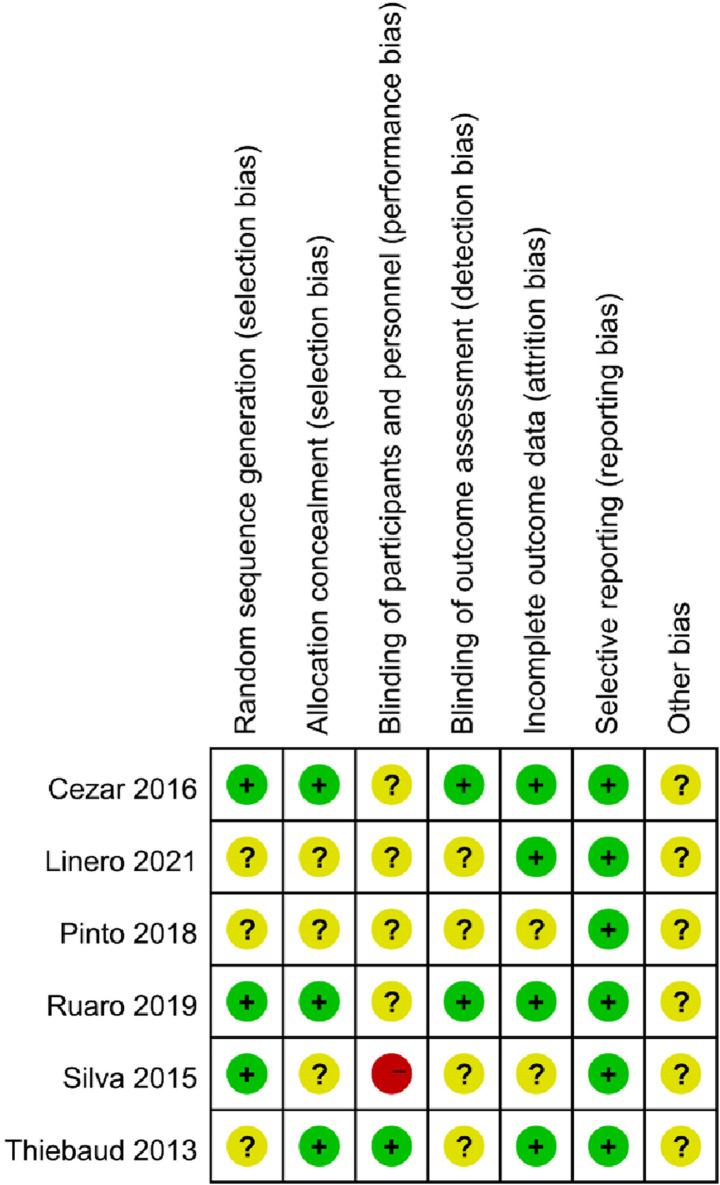
Risk profile of bias in included studies.

Figure 2 is the risk of bias assessment diagram. Figure 3 is the general summary risk of bias diagram: randomization of sequencing has 50% studies at low risk, 50% at unknown risk and 0% at high risk. The allocation selection method is observed with 50% studies at low risk, 50% at unclear risk and 0% at high risk. The blinding of participants had 17% studies at low risk, 66% at unknown risk, and 17% at high risk. The blinding of experiment operators was evaluated with 33% studies at low risk, 67% at unknown risk, and 0% at high risk. The incomplete reporting of the outcome data was 67% studies at low risk, 33% at unknown risk, and 0% at high risk. Selective reporting of results is at low risk, where no unclear risk and high risk was reported. The other sources of bias are unclear.

### 3.4 Meta analysis

#### 3.4.1 Synthesized results

**FIGURE 4:**
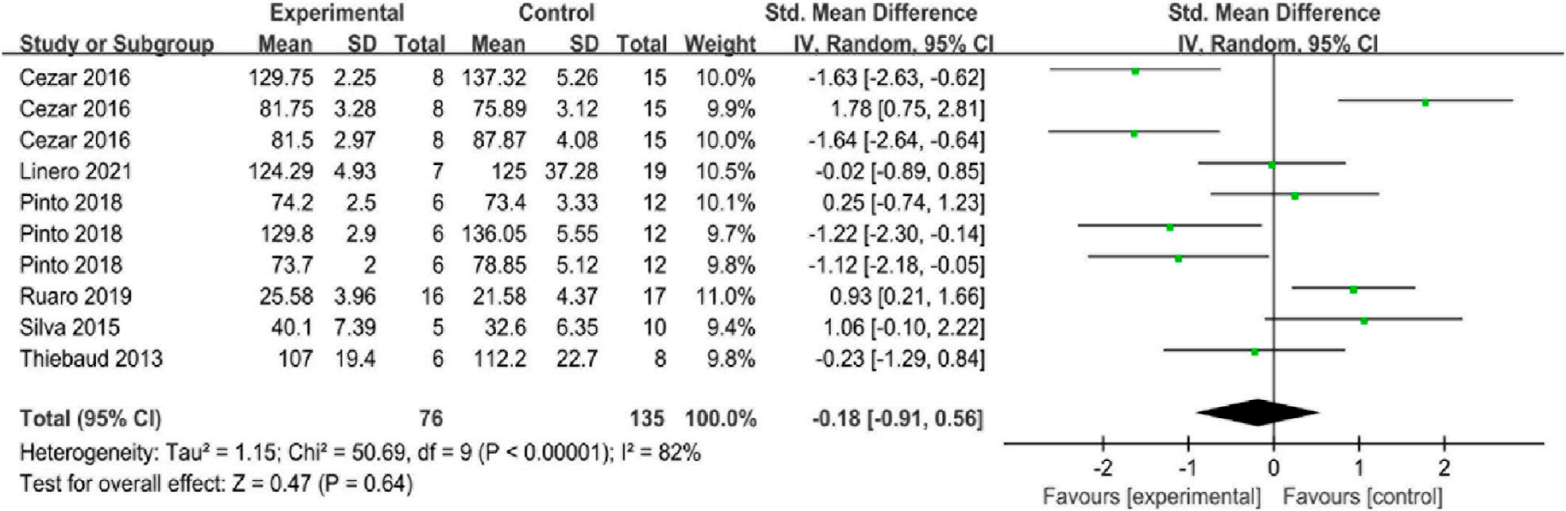
Forest plot for comprehensive analysis of included documents.

The overall effect test was conducted on six articles and 10 groups of data included in this study. The forest plot in Figure 4 showed that the heterogeneity among the included studies was high (I^2^ = 82%, *p* < 0.05) regarding all the included health indicators, so the random effects model was adopted. The combined effect size was SMD = −0.18 (95%CI: −0.91 to 0.56; *p* > 0.05), and the standard mean difference between the two groups was not significantly different.

#### 3.4.2 The maximum dynamic force of 1RM

**FIGURE 5:**
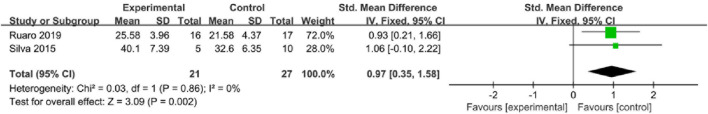
Forest plot of the maximum dynamic force of 1RM in the included literature.

Two studies reported the maximum dynamic force of 1RM, with a total of 48 subjects. As shown in Figure 5, I^2^ = 0% (*p* > 0.05), indicating that there was no heterogeneity between the two studies, so the fixed-effect model was selected for the 1RM analysis. The results showed that BFR could significantly enhance the maximum dynamic force of 1RM in middle-aged and elderly women, and the intervention effect was better than that of the control group, with SMD = 0.97 (95%CI: 0.35 to 1.58; *p* < 0.05), indicating that the experimental group was better than the control group in enhancing the maximum dynamic force of 1RM.

#### 3.4.3 Leg compression force

**FIGURE 6:**
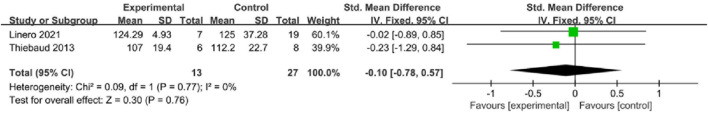
Forest plot of leg compression force in the included literature.

The leg compression force was reported in two studies with a total of 40 subjects. As shown in Figure 6, I^2^ = 0% (*p* > 0.05), indicating that there was no heterogeneity between the two studies, so the fixed-effect model was selected for leg compression force analysis. Results of meta-analysis showed that SMD = −0.10 (95%CI: −0.78 to 0.57; *p* > 0.05), which indicates that the mean difference between the two groups was not significantly different.

#### 3.4.4 Heart rate

**FIGURE 7:**
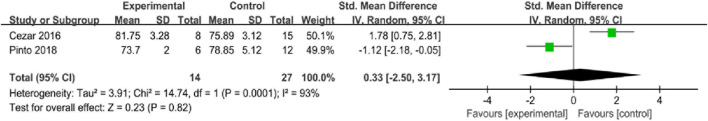
Heart rate forest plot of included references.

Two studies examined the effects of flow-restricted training on heart rate, involving a total of 41 subjects. As shown in Figure 7, heavy heterogeneity between the two studies was found, where I^2^ = 93% (*p* < 0.1), therefore the random effects model was selected for analysis. The results of meta-analysis showed that SMD = 0.33 (95%CI: −2.50 to 3.17; *p* > 0.05), indicating that there was no significant different impact on heart rate between the two groups.

When assigning medium-high intensity resistance training without BFR as the control group, it was observed that *p* ≤ 0.1, I^2^ = 74% (Appendix 1). Meanwhile, when assigning the group without any training as the control group, it was found that *p* ≤ 0.1, I^2^ = 96% (Appendix 2). The heterogeneity can be induced by the differences in the subjects selection, different study design, and the measurement methods and time.

#### 3.4.5 Blood pressure

**FIGURE 8:**
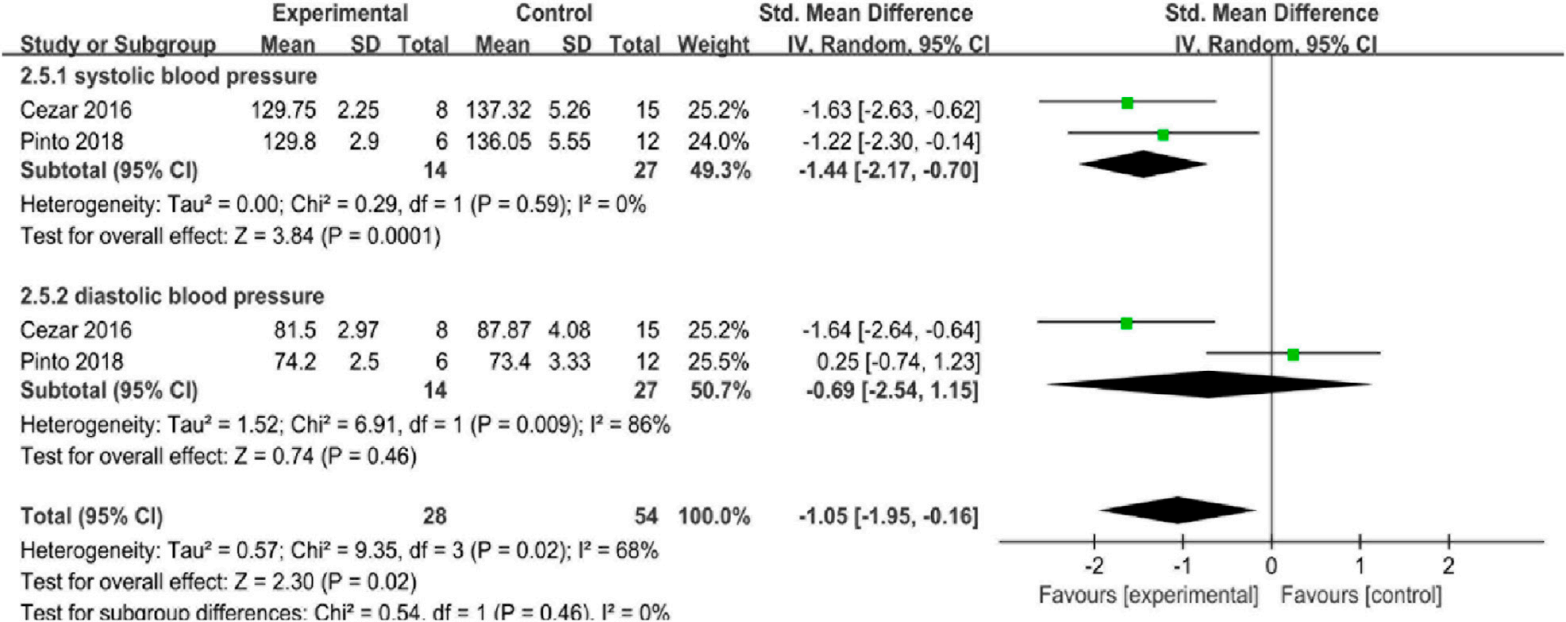
Forest plot of blood pressure in included literature.

Two studies examined the effect of BFR training on blood pressure, involving a total of 41 subjects. As shown in Figure 8, I^2^ = 68% (*p* = 0.02), with significant heterogeneity among the four studies being observed, therefore, the random effects model is applied. Results of meta-analysis showed a significant overall effect size SMD = −1.05 (95%CI: −1.95 to −0.16; *p* < 0.05), which indicates that BFR training can significantly reduce blood pressure of middle-aged and elderly women, and its effect is better than that of control group.

The heterogeneity of systolic blood pressure (SBP) was I^2^ = 0% (*p* = 0.59), where SMD = −1.44 (95%CI: −2.17 to −0.70; *p* < 0.05), indicating that BFR training can significantly reduce SBP of middle-aged and elderly women, and the experimental group is better than the control group in reducing SBP. Diastolic blood pressure (DBP), I^2^ = 86% (*p* = 0.009), SMD = −0.69 (95%CI: −2.54 to 1.15; *p* > 0.05), indicating that the experimental group and the control group might have similar effects on DBP, therefore, the significant overall effect of BFR on blood pressure majorly relies on its impact on the changes of SBP.

#### 3.4.6 Sensitivity analyses

In the Review Manager 5.4 software, after removing the included data one by one in the meta-analysis of the merging effect of 10 groups of data, it was found that the heterogeneity of the results after the elimination did not change significantly, which failed to change the heterogeneity of the merging effect analysis. Since only two literature are included in Figure 7 for analysis, it is not possible to conduct heterogeneity analysis if one of them is excluded.

#### 3.4.7 Reporting biases

**FIGURE 9:**
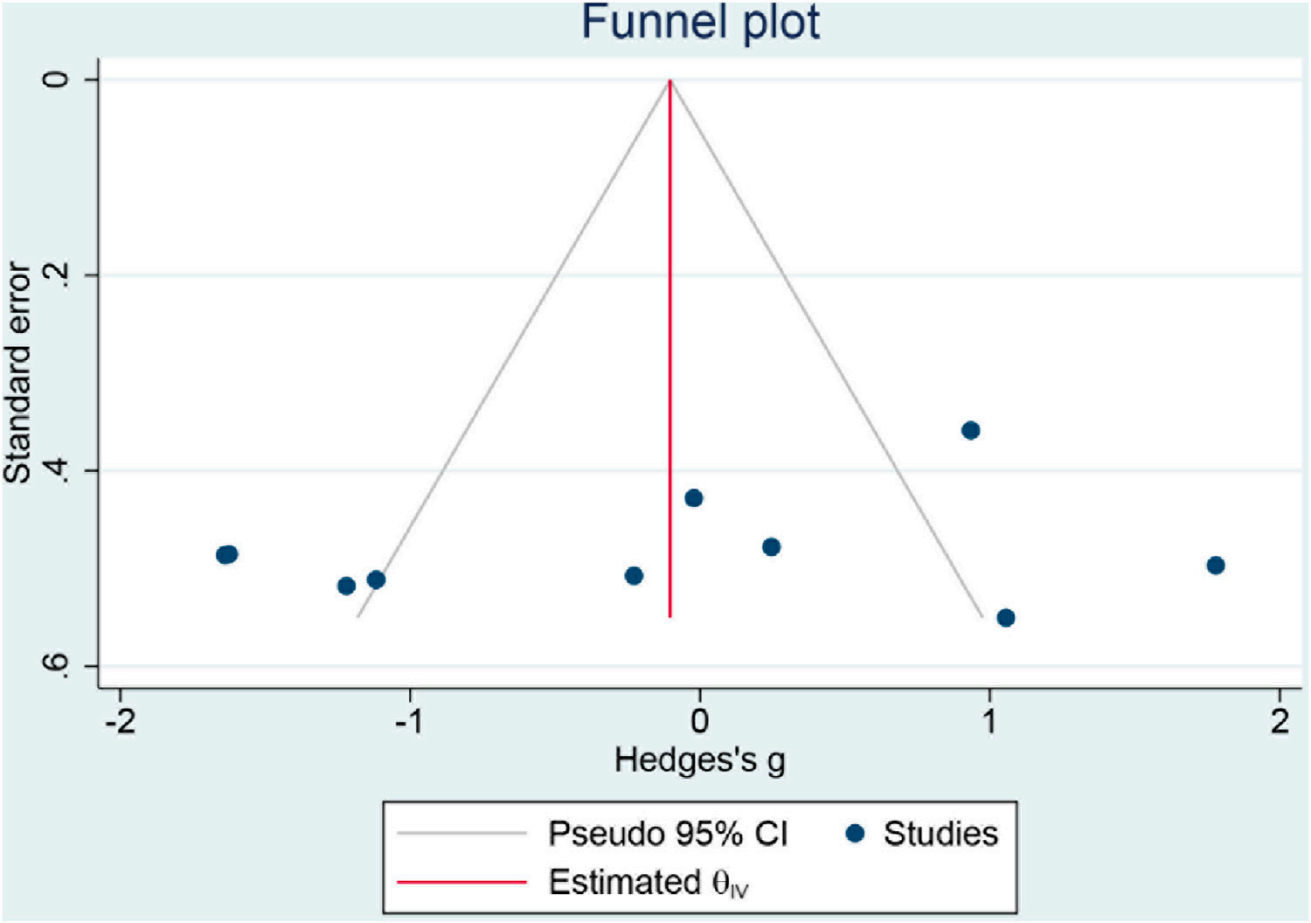
Funnel plot of risk of reporting biases.

The data were incorporated into Stata17 and Egger test was adopted to test the publication bias, which showed no significant publication bias with *p* > 0.05. Figure 9 shows the funnel plot of reporting bias risk.

## 4 Discussion

BFR training refers to the use of specific tools to add pressure the outside of the limb to achieve the purpose of obstructing blood flow within a certain range, combining with low-intensity exercise (Kraemer and Ratamess, 2004), inducing muscle reactive hyperemia (Pan Yanrong et al., 2021), promote the increase of protein synthesis and growth hormone secretion (Ronghai, 2019), which improves skeletal muscle adaptability, and then increases muscle thickness and cross-sectional area (Ferraz et al., 2018), and ultimately improves muscle strength (Madarame et al., 2011). Flow-restricted training can inhibit the expression of FOXO3A protein, thereby reducing the expression of Atrogin-1 and MuRF-1, and alleviate muscle atrophy (Sandri et al., 2004; Mammucari et al., 2007; Manini et al., 2011). When limiting blood flow, the training limb is suffering from ischemic stress, the peripheral artery metabolic pressure increases, and accompanied by higher active potential, resulting in a phenomenon similar to fatigue, and then, recruit type II fast-twitch muscle fiber, alternating the muscle fiber percentage, and stimulate muscle hypertrophy (Yasuda et al., 2005).

However, while improving muscle strength, BFR training will lead to relative ischemia and hypoxia of limbs (Yasuda et al., 2010), increase the release of K^+^ and Na^+^ to the extra cellular matrix (Franz et al., 2020), accumulation of metabolites such as lactic acid (Teixeira et al., 2018), acidosis, excessive metabolic pressure, and prone to thrombosis, abnormal cardiovascular reactions, muscle fatigue and injury. Due to the decline of body function, middle-aged and elderly people are at risk of various cardiovascular diseases (Yu Wei and Liu, 2022). For instance, physically inactive and attenuated muscle metabolism might be the risk factors for the developing and progression of cardiovascular or metabolic disorders.

Although BFR training has some potential risks, there are still a large number of experts taking this training method for clinical practice and research. After collecting the adverse events reported in the study, only two adverse events were reported (Ozawa et al., 2015; Harper et al., 2019), and fewer adverse events were reported than resistance training in the same study, and the intervention appears to be safe compared to high-intensity training. After a 6-month resistance training program, older women gain only one-fifth of the muscle strength of older men, and can gain more benefits from training by increasing the volume and intensity of exercise training, but for middle-aged and older women, high-intensity and high-load exercise is difficult to adapt (Hunter et al., 2004). Thiebaud et al. found that moderate to high intensity elastic band (EB) training and low intensity EB training were similar to BFR in improving strength, lean muscle mass and muscle thickness (Thiebaud et al., 2013). Vechin et al. found that both training regimens can effectively increase the quadriceps muscle strength and 1RM of the leg press, which is a feasible alternative exercise for people who cannot use high intensity exercise (Vechin et al., 2015). MÃ¢nica et al. found that BFR combined with low-intensity aerobic exercise had similar anti-inflammatory effects in older women with hypertension, and that BFR combined with low-intensity exercise promoted better immune responses and adaptation than low-intensity regimens (Mânica et al., 2020). These studies further confirm that BFR training is an important alternative to high-intensity resistance training and an effective training method for promoting bone health and muscle strength. BFR training is feasible for middle-aged and elderly women, but there are few studies on the optimal duration, intensity and flow-restriction tools of BRF training, and it is necessary to design appropriate training programs according to each person’s physical condition in the specific implementation process.

According to Figure 8, it appears that the overall effect size of quantitatively combined synthesis of SBP and DBP was improved with SMD = −1.05 (95%CI: −1.95 to −0.16; *p* < 0.05). Though blood flow restriction did not improve DBP compared to the control group by itself, where SMD = −0.69 (95%CI: −2.54 to 1.15; *p* > 0.05), and it showed that the SBP was downregulated after blood flow restriction training with SMD = −1.44 (95%CI: −2.17 to −0.70; *p* < 0.05). American Heart Association (AHA) and global healthcare practitioners recognize elevated SBP and DBP are among the risk factors of developing cardiac artery diseases (CAD) and cardiovascular diseases (CVD) (Price et al., 2019; Naidu et al., 2022; Kittleson et al., 2023). In this study, it can be observed that blood flow restriction training intervention has beneficial effect on the regulation of blood pressure, especially through down-regulating SBP, probably through enhancing eNOS synthesis and promoting the secretion of nitric oxide (NO) to the blood vessels smooth muscle endothelial cells (Li et al., 2020; Bao et al., 2022; Cai and Chen, 2022; Wu et al., 2022). Therefore, it is recommended for the elderly to practice blood flow restriction training under appropriate workload, which can be helpful in maintaining their regular blood pressure and have potential benefits in decreasing CAD and CVD risk factors.

This study investigated whether BFR training can effectively promote the physical health of middle-aged and elderly women. A total of six randomized controlled trial studies with middle-aged and elderly women were summarized, and 129 elderly women aged over 55 were included. The results of meta-analysis showed that flow-restricted training had significant effects on the improvement of muscle strength and reduction of SBP in middle-aged and elderly women, while there was no significant difference in heart rate and DBP. According to the included literature, the included subjects of their experiment were osteoporosis patients, hypertension patients and healthy elderly women, and the ethnics of population were diverse.

The muscle strength of middle-aged and elderly people will decrease with age (Genping, 2019), and the decline of muscle mass can lead to the osteoporosis and fall risk of elderly and middle-aged people (Ma et al., 2020). The muscle strength increases after BFR training (Yasuda et al., 2016; Early et al., 2020). After menopause, changes in hormone levels in the body will lead to decreased muscle strength, osteoporosis and high blood pressure (Li, 2022). Therefore, this study further systematically analyzed middle-aged and elderly women to clarify the health promotion effect of BFR, and through BFR training, middle-aged and elderly women can carry out physical exercise in a safe and efficient way. The results of this study are similar to those of previous studies in strengthening muscle strength (Slysz et al., 2016; Hughes et al., 2017; Liu Haoyan et al., 2023). Compared with previous studies on heart rate and blood pressure, there were some differences (Fei, 2016; Zhang et al., 2022). Wang Fang (Fei, 2016) reported a significant decrease in systolic blood pressure, but no significant difference in diastolic blood pressure compared with control group. However, Zhang et al. (2022) reported that BFR training can cause a sharp increase in heart rate and blood pressure in the elderly.

In the study (Patterson et al., 2019), the relevant suggestions about the safety of BFR training are put forward for BFR frequency, load, restriction time, cuff pressure, etc., but in the specific BFR training process, it is still necessary to be adjusted according to the different conditions of the subjects. Through literature review, it was found that there were great differences in the settings of these in the existing studies, and there were too few literature within the recommended range and in line with other inclusion criteria of this study. Therefore, the inclusion criteria of this study did not put forward restrictions on the cuff pressure value and time of the experimental BFR group. Therefore, BFR training should be treated with caution to improve the safety and effectiveness of BFR training as a form of exercise. In the future, the design and control factors of BFR training for different populations still need to be further explored, and the added pressure and training load that affect the actual BFR should be fully considered, and a safe and reasonable training scheme should be designed according to individuals’ needs.

### 4.1 Innovation

This study is the first meta-analysis to explore the effect of BFR training on health promotion in middle-aged and elderly women. All randomized controlled trials were included, and the sources of heterogeneity were carefully analyzed.

### 4.2 Limitations

The number of included literature was small, with only six RCTs, and some literature were of average quality. This study searched multiple databases in all language types, but the number of relevant studies obtained was small, and some articles were excluded because it was difficult to extract the required data, and too few literature were included in each outcome indicator. In the future, we can further increase the number randomized double-blind controlled trial studies on BFR training in middle-aged and elderly women. The heterogeneity of both heart rate and DBP was high, and the source of heterogeneity was explored. Differences in the health status of subjects, baseline measurement, experimental design and the different exercise type and compression zone might be the source of heterogeneity in this paper. The time and region of included studies might have different impact on the research results.

## 5 Conclusion

BFR training has a significant effect on improving and reducing blood pressure in middle-aged and elderly women. Middle-aged and elderly women can be recommended with BFR exercise to improve their health status. There is no significant different effect been observed in regard of improving heart rate and leg pressure strength, and further research is needed in the future.

## Data Availability

The original contributions presented in the study are included in the article/Supplementary Material, further inquiries can be directed to the corresponding authors.

## Abbreviations

BFR, blood flow restriction; RCT, randomized controlled trials; M, Mean; SD, Standard Deviation; SMD, standard mean differences; RM, repetition maximum; SBP, systolic blood pressure; DBP, diastolic blood pressure.
